# Supplementary material for: Fine manipulation of sound via lossy metamaterials with independent and arbitrary reflection amplitude and phase
Source: Nat Commun. 2018 Apr 24;9:1632. doi: 10.1038/s41467-018-04103-0 (PMC5915438; doi:10.1038/s41467-018-04103-0)
Supplement: Supplementary file 1 — Supplementary Information [file 41467_2018_4103_MOESM1_ESM.pdf]

Supplementary Information

Fine manipulation of sound via lossy metamaterials with independent and arbitrary reflection amplitude and phase

Zhu *et al.*

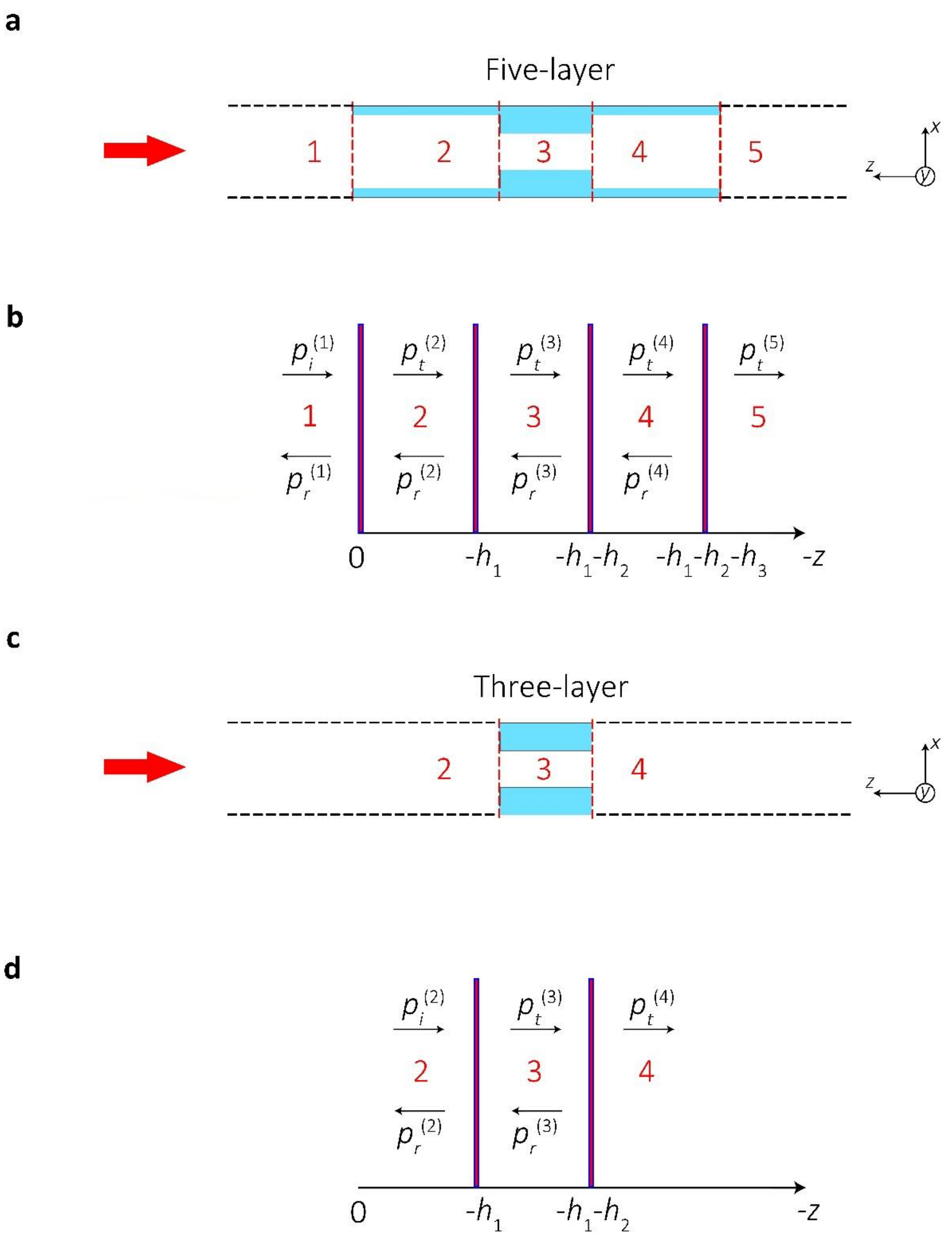

Supplementary Figure 1 | Illustration of the analytical model. (**a**) The lossy acoustic metamaterial (LAM) unit-cell can be regarded as a five-layer structure. (**b**) The analytical equivalence of the five-layer structure. (**c**) At the extreme case of $\beta = 1$, the LAM unit-cell is simplified into a three-layer structure. (**d**) The analytical equivalence of the three-layer structure.

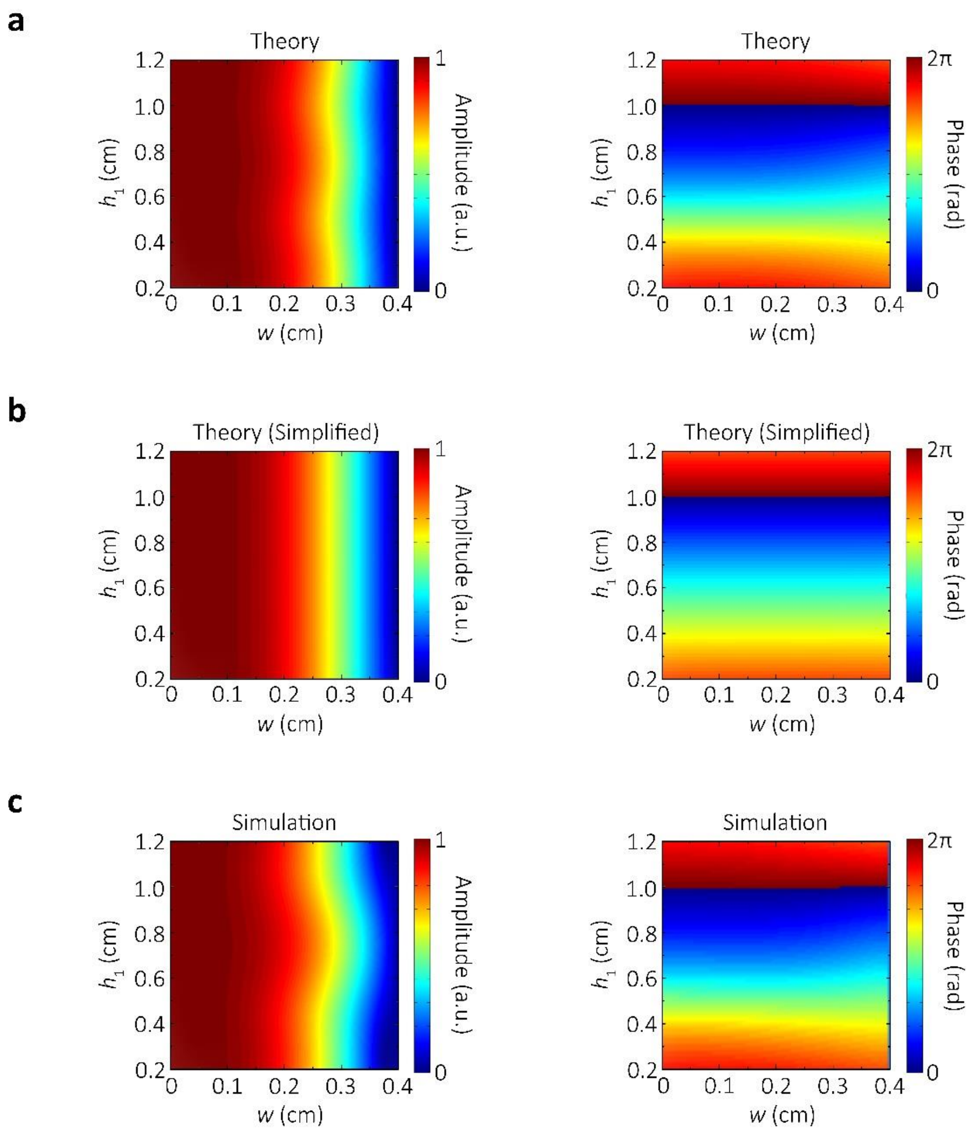

Supplementary Figure 2 | Analytical and numerical calculations of reflection amplitude A **and phase** ϕ**.** (**a**) A and ϕ calculated by Supplementary Eqs. (29) and (30) for different parameters of h_1 and w, when $\beta = 0.8$ and $h = 2\text{cm}$. (**b**) A and ϕ calculated by Supplementary Eqs. (37) and (39) for different parameters of h_1 and w, when $\beta = 1$. (**c**) Numerical calculations of A and ϕ for different parameters of h_1 and w, when $\beta = 0.8$ and $h = 2\text{cm}$.

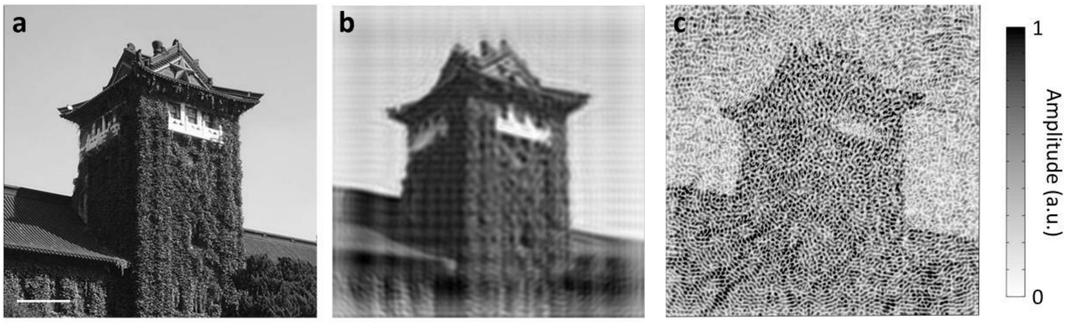

Supplementary Figure 3 | Simulations for a complicated image. (**a**) The target image with a complex amplitude distribution. (Scale bar, 20cm) (**b**) The generated holographic image via the amplitude-phase-modulation (APM) method. (**c**) The generated holographic image via the phase-modulation (PM) method. Rights for use of this image are from Nanjing University. All rights reserved.

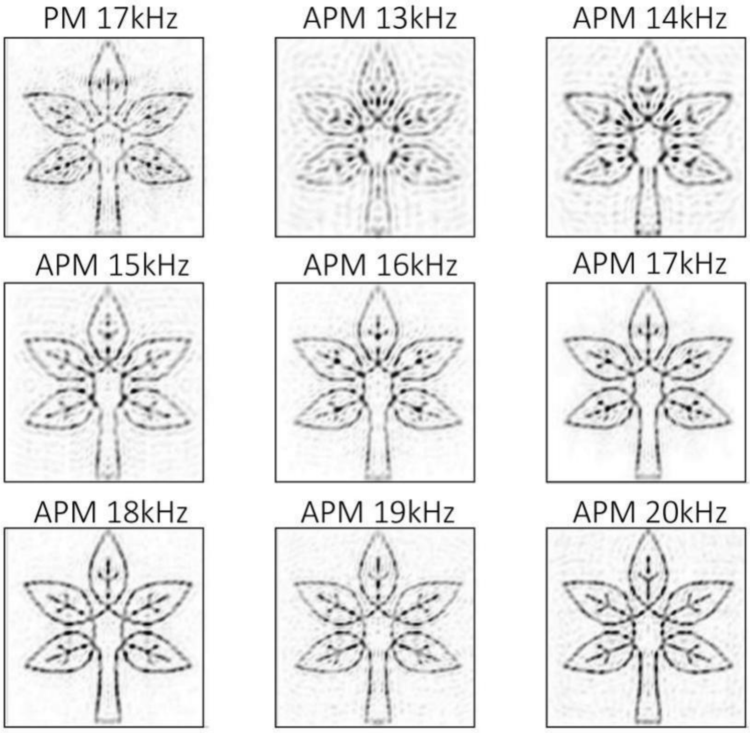

Supplementary Figure 4 | The holographic images calculated by the PM or APM method at different frequencies. Rights for use of this image are from Dreamstime

(www.dreamstime.com). All rights reserved.

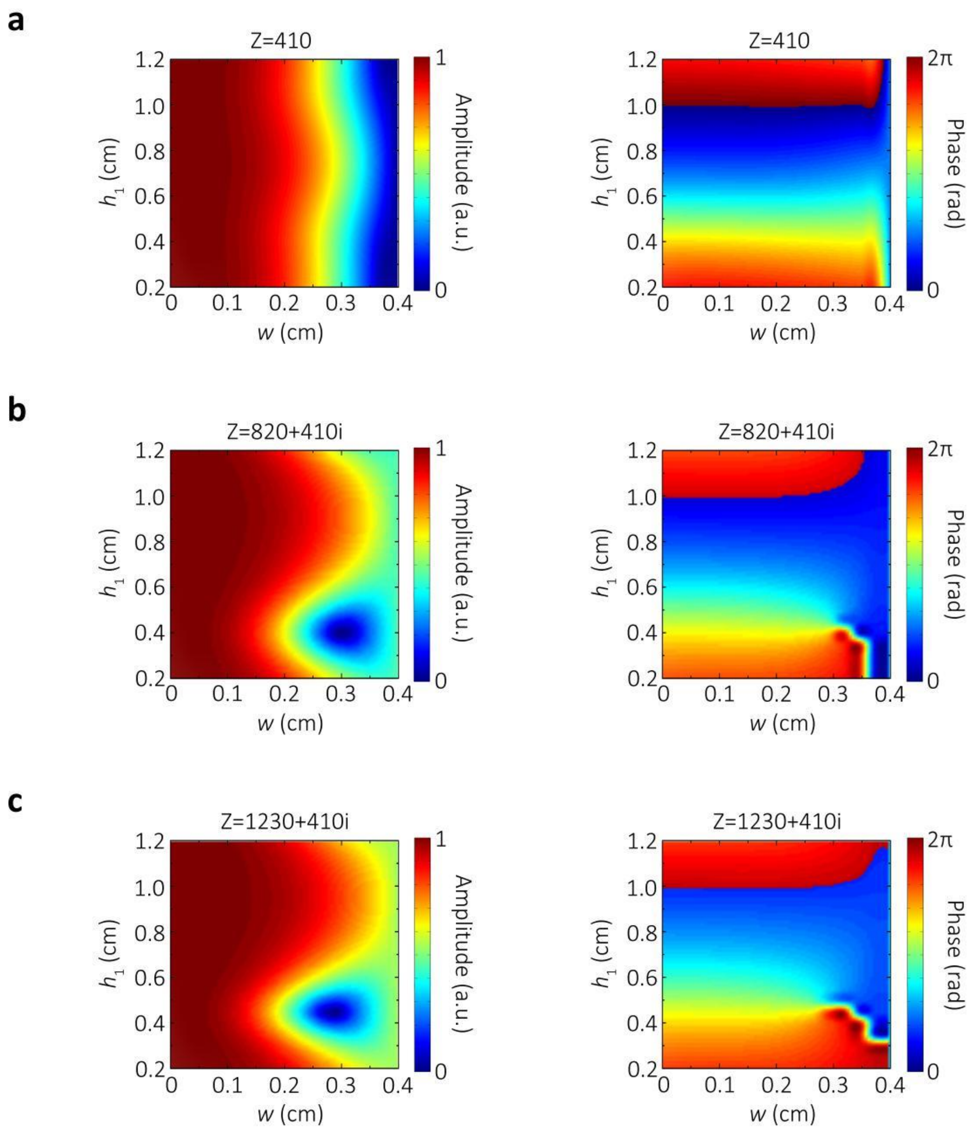

Supplementary Figure 5 | The calculated reflection amplitude and phase for different back impedances. (**a**) Z=410N·s·m^{-3}, (**b**) Z=820+410iN·s·m^{-3}, (**c**) Z=1230+410iN·s·m^{-3}.

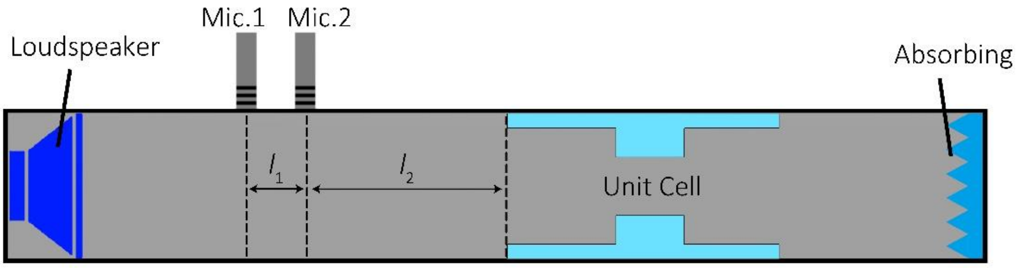

Supplementary Figure 6 | Schematic of the acoustic impedance tube. The loudspeaker and a pair of microphones (Mic. 1 and Mic. 2) are set in front of the unit cell for measuring the reflection amplitude and phase. The transmitted sound is absorbed by the absorbing back wall.

Supplementary Note 1. Analytical derivation of reflection amplitude and phase.

Supplementary Fig. 1(a) shows a general condition ($\beta = d/D < 1$), where the unit-cell is divided into five parts with different cross sections, defined as Medium 1 to Medium 5, respectively. The media have different acoustic impedances. As shown in Supplementary Fig. 1(b), $p_i^{(1)}$ denotes the incident waves, while $p_t^{(n)}$ and $p_r^{(n)}$ are the transmitted and reflected waves in the nth medium. The widths of Media 2, 3, and 4 are h_1, h_2, and h_3, respectively. The acoustic pressures in Media 1-5 are expressed as follows

$$p_i^{(1)} = p_{0i}^{(1)} \exp(ik_0 z),\ p_r^{(1)} = p_{0r}^{(1)} \exp(-ik_0 z), \tag{1}$$

$$p_t^{(2)} = p_{0t}^{(2)} \exp(ik_0 z),\ p_r^{(2)} = p_{0r}^{(2)} \exp(-ik_0 z), \tag{2}$$

$$p_t^{(3)} = p_{0t}^{(3)} \exp[ik_0(z+h_1)],\ p_r^{(3)} = p_{0r}^{(3)} \exp[-ik_0(z+h_1)], \tag{3}$$

$$p_t^{(4)} = p_{0t}^{(4)} \exp[ik_0(z+h_1+h_2)],\ p_r^{(4)} = p_{0r}^{(4)} \exp[-ik_0(z+h_1+h_2)], \tag{4}$$

$$p_t^{(5)} = p_{0t}^{(5)} \exp[ik_0(z+h_1+h_2+h_3)]. \tag{5}$$

The boundary conditions at the four interfaces are expressed as

$$p_{0i}^{(1)} + p_{0r}^{(1)} = p_{0t}^{(2)} + p_{0r}^{(2)}, \tag{7}$$

$$\frac{p_{0i}^{(1)} - p_{0r}^{(1)}}{R_1} = \frac{p_{0t}^{(2)} - p_{0r}^{(2)}}{R_2}, \tag{8}$$

$$p_{0t}^{(2)} \exp(-ik_0 h_1) + p_{0r}^{(2)} \exp(ik_0 h_1) = p_{0t}^{(3)} + p_{0r}^{(3)}, \tag{9}$$

$$\frac{p_{0t}^{(2)} \exp(-ik_0 h_1) - p_{0r}^{(2)} \exp(ik_0 h_1)}{R_2} = \frac{p_{0t}^{(3)} - p_{0r}^{(3)}}{R_3}, \tag{10}$$

$$p_{0t}^{(3)} \exp(-ik_0 h_2) + p_{0r}^{(3)} \exp(ik_0 h_2) = p_{0t}^{(4)} + p_{0r}^{(4)}, \tag{11}$$

$$\frac{p_{0t}^{(3)} \exp(-ik_0 h_2) - p_{0r}^{(3)} \exp(ik_0 h_2)}{R_3} = \frac{p_{0t}^{(4)} - p_{0r}^{(4)}}{R_4}, \tag{12}$$

$$\frac{p_{0t}^{(4)}\exp(-ik_0h_3)-p_{0r}^{(4)}\exp(ik_0h_3)}{R_4}=\frac{p_{0t}^{(5)}}{R_5}, \tag{13}$$

$$p_{0t}^{(4)}\exp(-ik_0h_3)+p_{0r}^{(4)}\exp(ik_0h_3)=p_{0t}^{(5)}, \tag{14}$$

where R_1, R_2, R_3, R_4, and R_5 are the normalized impedances of Media 1-5 and are proportional to the cross section areas

$$R_1=R_5=1, \tag{15}$$

$$R_2=R_4=\beta^2, \tag{16}$$

$$R_3=(w/0.005)^2. \tag{17}$$

The relations of wave components in two neighboring media are characterized by

$$\begin{bmatrix} p_{0t}^{(2)} \\ p_{0r}^{(2)} \end{bmatrix}=Q_1\begin{bmatrix} p_{0i}^{(1)} \\ p_{0r}^{(1)} \end{bmatrix}, \tag{18}$$

$$\begin{bmatrix} p_{0t}^{(3)} \\ p_{0r}^{(3)} \end{bmatrix}=Q_2\begin{bmatrix} p_{0t}^{(2)} \\ p_{0r}^{(2)} \end{bmatrix}, \tag{19}$$

$$\begin{bmatrix} p_{0t}^{(4)} \\ p_{0r}^{(4)} \end{bmatrix}=Q_3\begin{bmatrix} p_{0t}^{(3)} \\ p_{0r}^{(3)} \end{bmatrix}, \tag{20}$$

$$\begin{bmatrix} p_{0t}^{(5)} \\ 0 \end{bmatrix}=Q_4\begin{bmatrix} p_{0t}^{(4)} \\ p_{0r}^{(4)} \end{bmatrix}, \tag{21}$$

where Q_1, Q_2, Q_3, and Q_4 are deduced from Supplementary Eqs. (7-14)

$$Q_1=\frac{1}{2}\begin{bmatrix} 1+R_{12}, & 1-R_{12} \\ 1-R_{12}, & 1+R_{12} \end{bmatrix}, \tag{22}$$

$$Q_2=\frac{1}{2}\begin{bmatrix} (1+R_{23})\exp(-ik_0h_1), & (1-R_{23})\exp(ik_0h_1) \\ (1-R_{23})\exp(-ik_0h_1), & (1+R_{23})\exp(ik_0h_1) \end{bmatrix}, \tag{23}$$

$$Q_3=\frac{1}{2}\begin{bmatrix} (1+R_{34})\exp(-ik_0h_2), & (1-R_{34})\exp(ik_0h_2) \\ (1-R_{34})\exp(-ik_0h_2), & (1+R_{34})\exp(ik_0h_2) \end{bmatrix}, \tag{24}$$

$$Q_4=\frac{1}{2}\begin{bmatrix} (1+R_{45})\exp(-ik_0h_3), & (1-R_{45})\exp(ik_0h_3) \\ (1-R_{45})\exp(-ik_0h_3), & (1+R_{45})\exp(ik_0h_3) \end{bmatrix}. \tag{25}$$

where the ratios of normalized impedances are denoted by

$$R_{12}=\frac{R_2}{R_1},R_{23}=\frac{R_3}{R_2},R_{34}=\frac{R_4}{R_3},R_{45}=\frac{R_5}{R_4}. \tag{26}$$

We finally obtain the relation of the incident, reflected, and transmitted waves for the unit-cell based on transfer matrix method

$$\begin{bmatrix} p_{0t}^{(5)} \\ 0 \end{bmatrix} = Q \begin{bmatrix} p_{0i}^{(1)} \\ p_{0r}^{(1)} \end{bmatrix}, \tag{27}$$

where Q is derived by

$$Q=\begin{bmatrix} q_{11} & q_{12} \\ q_{21} & q_{22} \end{bmatrix} = Q_4 Q_3 Q_2 Q_1 . \tag{28}$$

The normalized amplitude and phase of reflection (A and ϕ) can be calculated by

$$A=\left|\frac{p_{0r}^{(1)}}{p_{0i}^{(1)}}\right| = \left|\frac{q_{21}}{q_{22}}\right|, \tag{29}$$

$$\phi = \arg\left(\frac{q_{21}}{q_{22}}\right). \tag{30}$$

Supplementary Note 2. Reflection amplitude and phase at $\beta=1$.

At the extreme case of $\beta=1$, the five-layer structure is simplified to a three-layer structure, as shown in Supplementary Figs. 1c and 1d. In this case, the relation of the incident, reflected, and transmitted waves is

$$\begin{bmatrix} p_{0t}^{(4)} \\ 0 \end{bmatrix} = Q \begin{bmatrix} p_{0i}^{(2)} \\ p_{0r}^{(2)} \end{bmatrix}, \tag{31}$$

where Q is

$$Q=\begin{bmatrix} q'_{11}, & q'_{12} \\ q'_{21}, & q'_{22} \end{bmatrix} = Q_3 Q_2 , \tag{32}$$

and

$$Q_2 = \frac{1}{2}\begin{bmatrix} 1+R_{23}, & 1-R_{23} \\ 1-R_{23}, & 1+R_{23} \end{bmatrix}, \tag{33}$$

$$Q_3 = \frac{1}{2}\begin{bmatrix} (1+R_{34})\exp(-ik_0h_2), & (1-R_{34})\exp(ik_0h_2) \\ (1-R_{34})\exp(-ik_0h_2), & (1+R_{34})\exp(ik_0h_2) \end{bmatrix}, \tag{34}$$

The reflection amplitude is calculated by

$$A = \left|\frac{p_{0r}^{(1)}}{p_{0i}^{(1)}}\right| = \left|\frac{q'_{21}}{q'_{22}}\right|. \tag{35}$$

We can further obtain

$$A = \sqrt{1 - \frac{4}{4\cos^2 k_0h_2 + (R_{23} + 1/R_{23})^2 \sin^2 k_0h_2}}. \tag{36}$$

Supplementary Eq. (36) is agreed with the result in Supplementary Ref. 1. In our model, at the particular case of $h_2 = \lambda/4$ or $kh_2 = \pi/2$, we can obtain a simplified expression of reflection amplitude

$$A = \frac{d^4 - w^4}{d^4 + w^4}. \tag{37}$$

And the reflection phase is thus expressed by

$$\phi = \arg\left(\frac{q'_{21}}{q'_{22}}\right) - \frac{4\pi h_1}{\lambda}, \tag{38}$$

where the term $-4\pi h_1/\lambda$ is the phase delay in Medium 2. By combining above equations, we will get $q'_{21}/q'_{22} = (d^4 - w^4)/(d^4 + w^4)$, leading to the simplified expression of reflection phase

$$\phi = -\frac{4\pi h_1}{\lambda}. \tag{39}$$

Supplementary Note 3. Reflection amplitude and phase at $\beta < 1$.

As aforementioned, Supplementary Eqs. (37) and (39) are the exact solutions at

$\beta = 1$, which manifests a decoupling condition. However, in our proposed model, we focus on the more practical quasi-decoupling condition, where the structural parameters $\beta = 0.8$ and $h = n\lambda / 2$ $(n = 2, 3, 4, \cdots)$. Supplementary Fig. 2 shows the results of the simplified case that $\beta = 1$ and the practical case that $\beta = 0.8$ and $h = 2\text{cm}$. To be specific, Supplementary Fig. 2(a) gives the analytical results of the reflection amplitude A and phase ϕ, calculated by Supplementary Eqs. (29) and (30), when $\beta = 0.8$ and $h = 2\text{cm}$. Supplementary Fig. 2(b) gives the simplified calculations of A and ϕ by Supplementary Eqs. (37) and (39), when $\beta = 1$. Clearly, we observe a good agreement between the two results of the quasi-decoupling and decoupling cases. Supplementary Fig. 2(c) shows the numerical calculations of A and ϕ by a finite element solver with the parameters $\beta = 0.8$ and $h = 2\text{cm}$, which however has some discrepancy to the result of Supplementary Fig. 2(a). Such discrepancy is attributed to the fact that the size of unit cells (or pixels) is not deep-subwavelength. If we further increase the pixel density, the numerical simulation will be remarkably consistent with the analytical solution.

Supplementary Note 4. Calculation of the coupling coefficients $\overline{M}_{A(\phi),h_1(w)}$.

The coupling coefficients $\overline{M}_{A(\phi),h_1(w)}$ in the manuscript are calculated by

$$\overline{M}_{A(\phi),h_1(w)} = \overline{M}_{A(\phi),h_1(w)}(\beta, h) / \max[\overline{M}_{A(\phi),h_1(w)}(\beta, h)], \tag{40}$$

where

$$\overline{M}_{A,h_1}(\beta, h) = \int_0^{\beta D} \int_{0.1\lambda}^{0.6\lambda} M_{A,h_1} dw dh_1 = \int_0^{\beta D} \int_{0.1\lambda}^{0.6\lambda} \frac{\partial A}{\partial h_1} dw dh_1, \tag{41}$$

$$\overline{M}_{A,w}(\beta,h)=\int_0^{\beta D}\int_{0.1\lambda}^{0.6\lambda} M_{A,w}dwdh_1=\int_0^{\beta D}\int_{0.1\lambda}^{0.6\lambda}\frac{\partial A}{\partial w}dwdh_1, \tag{42}$$

$$\overline{M}_{\phi,h_1}(\beta,h)=\int_0^{\beta D}\int_{0.1\lambda}^{0.6\lambda} M_{\phi,h_1}dwdh_1=\int_0^{\beta D}\int_{0.1\lambda}^{0.6\lambda}\frac{\partial \phi}{\partial h_1}dwdh_1, \tag{43}$$

$$\overline{M}_{\phi,w}(\beta,h)=\int_0^{\beta D}\int_{0.1\lambda}^{0.6\lambda} M_{\phi,w}dwdh_1=\int_0^{\beta D}\int_{0.1\lambda}^{0.6\lambda}\frac{\partial \phi}{\partial w}dwdh_1 \tag{44}$$

with A and ϕ being calculated by Supplementary Eqs. (29) and (30).

Supplementary Note 5. Calculation of the correlation between two images.

The correlation for evaluating the similarity between two images is calculated by

$$\text{Correlation}=\frac{\sum_m\sum_n (A_{mn}-\overline{A})(B_{mn}-\overline{B})}{\sqrt{\left(\sum_m\sum_n (A_{mn}-\overline{A})^2\right)\left(\sum_m\sum_n (B_{mn}-\overline{B})^2\right)}}, \tag{45}$$

where *A* and *B* are the data matrices of the two images, and $\overline{A}$ and $\overline{B}$ are the mean values of the elements in the matrices *A* and *B*, respectively.

Supplementary Note 6. The method of measuring the reflection amplitude and phase.

By using an acoustic impedance tube shown in Supplementary Fig. 6, the reflectance factor $r=Ae^{i\varphi}$ can be obtained based on the transfer function method in Supplementary Ref. 2, as follows

$$r=\frac{H_{12}-e^{-ik_0l_1}}{e^{ik_0l_1}-H_{12}}e^{2ik(l_1+l_2)}, \tag{46}$$

where $H_{12}=p_2/p_1$ is the transfer function between the acoustic pressure measured by the Microphones 1 and 2 and processed by the Brüel&Kjær PULSE Multi-analyzer system. Then, the reflected amplitude and phase can be directly calculated from

$$A = |r|, \tag{47}$$

$$\phi = \arg(r). \tag{48}$$

Supplementary References

1. Kinsler, L. *Fundamentals of Acoustic* (Wiley, New York, 1982).

2. ISO, EN. 10534-2. Acoustics-Determination of sound absorption coefficient and impedance in impedance tubes-Part 2 (1998).
